# Improvement in lung function and functional capacity in morbidly obese women subjected to bariatric surgery

**DOI:** 10.6061/clinics/2018/e20

**Published:** 2018-02-22

**Authors:** Elaine Cristina de Campos, Fabiana Sobral Peixoto-Souza, Viviane Cristina Alves, Renata Basso-Vanelli, Marcela Barbalho-Moulim, Rafael Melillo Laurino-Neto, Dirceu Costa

**Affiliations:** I Programa de Pos-graduacao em Ciencias da Reabilitacao, Universidade Nove de Julho, Sao Paulo, SP, BR; II Fisioterapia, Universidade Federal de Sao Carlos, Sao Carlos, SP, BR; III Fisioterapia, Hospital Meridional, Cariacica, ES, BR; IV Complexo Hospitalar do Mandaqui, Centro Multidisciplinar para Tratamento Cirurgico da Obesidade Morbida, Sao Paulo, SP, BR

**Keywords:** Bariatric Surgery, Functional Capacity, Lung Function, Body Composition

## Abstract

**OBJECTIVE:**

To determine whether weight loss in women with morbid obesity subjected to bariatric surgery alters lung function, respiratory muscle strength, functional capacity and the level of habitual physical activity and to investigate the relationship between these variables and changes in both body composition and anthropometrics.

**METHODS:**

Twenty-four women with morbid obesity were evaluated with regard to lung function, respiratory muscle strength, functional capacity, body composition, anthropometrics and the level of habitual physical activity two weeks prior to and six months after bariatric surgery.

**RESULTS:**

Regarding lung function, mean increases of 160 mL in slow vital capacity, 550 mL in expiratory reserve volume, 290 mL in forced vital capacity and 250 mL in forced expiratory volume in the first second as well as a mean reduction of 490 mL in inspiratory capacity were found. Respiratory muscle strength increased by a mean of 10 cmH_2_O of maximum inspiratory pressure, and a 72-meter longer distance on the Incremental Shuttle Walk Test demonstrated that functional capacity also improved. Significant changes also occurred in anthropometric variables and body composition but not in the level of physical activity detected using the Baecke questionnaire, indicating that the participants remained sedentary. Moreover, correlations were found between the percentages of lean and fat mass and both inspiratory and expiratory reserve volumes.

**CONCLUSION:**

The present data suggest that changes in body composition and anthropometric variables exerted a direct influence on functional capacity and lung function in the women analyzed but exerted no influence on sedentarism, even after accentuated weight loss following bariatric surgery.

## INTRODUCTION

Obesity is a public health problem that affects alarming portions of the worldwide population. Estimates indicate that approximately 2.3 billion adults will be overweight in the upcoming years, with more than 700 million classified as obese 1. In Brazil, 56% of the population has excess weight, and 13% of these individuals are classified as obese 2.

Obesity is considered a significant risk factor for cardiovascular disease, type 2 diabetes, high blood pressure, dyslipidemia and neoplasms 3-5. Moreover, obesity significantly compromises respiratory mechanics and causes direct changes in lung volumes 6. Such breathing problems are mainly due to upward displacement of the diaphragm, with a consequent greater difficulty in lung expansion, leading to a reduction in expiratory reserve volume as well as an increase in respiratory muscle work 7,8. Thus, lung function and respiratory muscle strength are receiving greater attention in studies on obesity 9-11.

Obesity causes direct alterations in lung volumes and complacency and leads to an altered ventilation/perfusion (V/Q) ratio, retention of carbon dioxide (CO_2_), increased resistance to air flow and an increase in the respiratory rate, with consequent ventilatory limitations, especially during exertion 12.

Compromised functional capacity due to obesity can be determined using a simple test, such as the Incremental Shuttle Walk Test (ISWT) 13, which has been employed in different populations to detect possible changes in physical fitness 14. As an incremental test, however, the ISWT is utilized with certain limitations. Despite findings of the applicability of this type of test in other populations 15, evidence regarding its use in individuals with obesity is scarce. Furthermore, there are no reports comparing functional capacity in obese individuals evaluated with the ISWT before and after bariatric surgery.

Some findings have indicated that the considerable weight loss as a result of bariatric surgery can reverse changes in respiratory mechanics in obese individuals, thereby improving tolerance to physical exercise and enhancing aerobic capacity 16,17. It is therefore important to investigate the respiratory and mechanical changes invoked by obesity and their associations with body composition, physical performance and functional capacity as well as the relationship with weight loss after bariatric surgery 18,19.

Thus, the aim of the present study was to determine whether weight loss in women with morbid obesity subjected to bariatric surgery alters lung function, respiratory muscle strength, functional capacity and the level of habitual physical activity. A further aim was to investigate the relationship between these variables and changes in both body composition and anthropometrics.

## MATERIALS AND METHODS

A prospective study was conducted to evaluate physical capacity and lung function in women with morbid obesity subjected to bariatric surgery. This study received approval from the Ethics Committee of Universidade Nove de Julho (process number 525.906/2014).

Female gender was chosen due to the higher number of female bariatric surgery candidates than male candidates at the study hospital. Women with morbid obesity were recruited from the Multidisciplinary Center for the Surgical Treatment of Morbid Obesity of the Mandaqui Hospital Complex located in São Paulo, Brazil. Evaluations were performed at the Respiratory Functional Assessment Laboratory of Universidade Nove de Julho in the preoperative (two weeks prior to surgery) and post-operative (six months after surgery) phases.

The inclusion criteria were female gender, age 18 years or older, body mass index (BMI) between 40 and 55 kg/m^2^, capacity to perform the proposed tests, awaiting bariatric surgery, clinical stability and agreement to participate through a signed statement of informed consent. The exclusion criteria were the presence of orthopedic or neurological conditions that impeded participation in a physical exercise program, acute or chronic lung disease, respiratory infection in the previous five weeks, smoking, uncontrolled arterial hypertension, obstructive lung conditions based on the criteria of the Global Initiative for Chronic Obstructive Lung Disease (GOLD) 20 or a restrictive lung condition based on a history of restrictive disease and confirmed by spirometry according to the criteria of the Brazilian Thoracic Association 21.

### Lung function

Lung function was assessed using a computerized ultrasound spirometer with a flow sensor (Easy-One, NDD^®^, Medizintechnik, Switzerland) following the norms established by the American Thoracic Society (ATS) 22. The data are expressed as the percentage of predicted values established for the Brazilian population 23. The test consisted of maximum inspiration and expiration until three reproducible maneuvers were achieved, as recommended by the ATS 22. All participants remained seated with a nose clip in place and performed each of the three maneuvers three times: 1) slow vital capacity (SVC), which resulted in value for SVC as well as the expiratory reserve volume (ERV), inspiratory reserve volume (IRV) and inspiratory capacity (IC); 2) forced vital capacity (FVC), which resulted in values for FVC, forced expiratory volume in the first second (FEV_1_) and the FEV_1_/FVC ratio; and 3) maximum voluntary ventilation (MVV) 22.

### Respiratory muscle strength

Respiratory muscle strength was measured using an analog manometer (Wika^®^, GA, USA) with an operating range of -300 to 300 cmH_2_0 and equipped with a rigid plastic mouthpiece adapter containing an orifice with an internal diameter of two millimeters, which served as a relief valve for oral pressure. The maximum inspiratory and expiratory pressures (MIP and MEP, respectively) were determined with the patient seated and wearing a nose clip. MIP was determined using the maximum inspiration maneuver beginning with maximum expiration; MEP was determined using the maximum expiration maneuver beginning with maximum inspiration 22,24. Each of these maneuvers was sustained for at least one second and repeated three to eight times. The highest value was accepted, provided that it did not exceed the next highest value by more than 10%. The equation proposed by Neder et al. 25 was used to calculate the predicted MIP and MEP.

### Functional capacity

Functional capacity was determined using the ISWT, which is a reliable, easy-to-administer, maximum exercise measure. The test was supervised by a trained researcher and performed on a ten-meter track along a corridor with two cones marking the beginning and the end 29. Prior to the test, the participant remained seated, and the following variables were monitored: blood pressure using a duly calibrated mercury column sphygmomanometer with a cuff appropriate for the arm circumference of the patients, heart rate using a pulse meter (Polar^®^, NY, USA), peripheral oxygen saturation (SpO_2_) using a pulse oximeter (Nonin^®^, NY, USA), shortness-of-breath (Borg Scale) and lower-limb exertion (Borg scale).

The test was explained in a standardized manner and demonstrated individually. The participants were instructed to walk at a constant pace, with the aim of reaching one of the cones upon hearing a recorded signal and to continue walking until they felt unable to maintain the required pace without becoming excessively out of breath. Heart rate, SpO_2_, shortness-of-breath (Borg scale) and lower-limb exertion (Borg scale) were monitored throughout the test. Blood pressure and breathing rate were determined at the end of each test. To minimize the learning effect, the test was performed twice, with a 30-minute rest period between to allow the vital signs to return to baseline levels 29. The longer distance travelled was considered for the purpose of analysis. The predicted distance was also calculated using the formula proposed in a previous study 19, in which the weight and height of each individual were taken into account.

### Anthropometric evaluation

BMI was calculated (kg/m^2^). Body mass was also used to calculate the percentage of excess weight based on the ideal weight defined by the Metropolitan Life Foundation and the Multi-Society Brazilian Consensus on Surgery for Obesity 26. Neck circumference (NC) was measured at the height of the cricoid cartilage 27. Waist circumference (WC) was measured at the midpoint between the lower edge of the last rib and upper edge of the iliac crest, and hip circumference was measured at the height of the greater trochanter of the femur 28. These measurements are expressed in centimeters (cm).

### Body composition

Body composition was determined using a magnetic bioimpedance device (BIODYNAMICS MODEL 450; Biodynamics Corporation, Seattle, WA, USA). With the participant in the supine position, four electrodes were placed at the extremities of the body: two on the dorsum of the hands and two on the dorsum of the feet. An alternating current at a frequency of 50 kHz was applied to the input electrodes, and body impedance was recorded based on the drop in voltage through the body to the output electrodes to determine the main variables: fat mass, lean mass, percentage of fat mass and percentage of lean mass.

### Physical activity questionnaire

Physical activity was evaluated using the Baecke questionnaire modified for epidemiological studies 29, which is a practical, fast and easy-to-understand measure 30. This qualitative-quantitative questionnaire is used to investigate habitual physical activity in the previous 12 months using eight items that address physical exercise in leisure (PEL – four items on the intensity and frequency of the practice of sports or physical exercise) and leisure locomotion activities (LLA – time spent watching television or riding a bicycle during leisure). Each item has a scale ranging from 1 to 5 points, with higher scores denoting a higher level of activity. A total score of less than 8 points denotes a sedentary lifestyle.

### Data analysis

The Shapiro-Wilk test was used to determine the distribution (normal or non-normal) of the data. A paired t-test was used for comparison of anthropometric variables, body composition, spirometric variables, respiratory muscle strength and functional capacity before and after surgery (intra-group). The Wilcoxon test was employed for nonparametric data referring to physical activity. Pearson’s and Spearman’s correlation coefficients were calculated for parametric and nonparametric variables, respectively, and interpreted as follows: <0.3 = weak correlation, 0.3 to 0.7 = moderate correlation and >0.7 = strong correlation. Data were analyzed using the BioEstat program, version 5.0, with the level of significance set to 5% (*p*≤0.05).

The sample was calculated based on previous studies addressing lung function, respiratory muscle strength and functional capacity in obese and non-obese individuals following bariatric surgery 18,19. Considering an 80% test power and an alpha error of 0.05, the sample size was determined to be 24 volunteers.

## RESULTS

**Figure 1:**
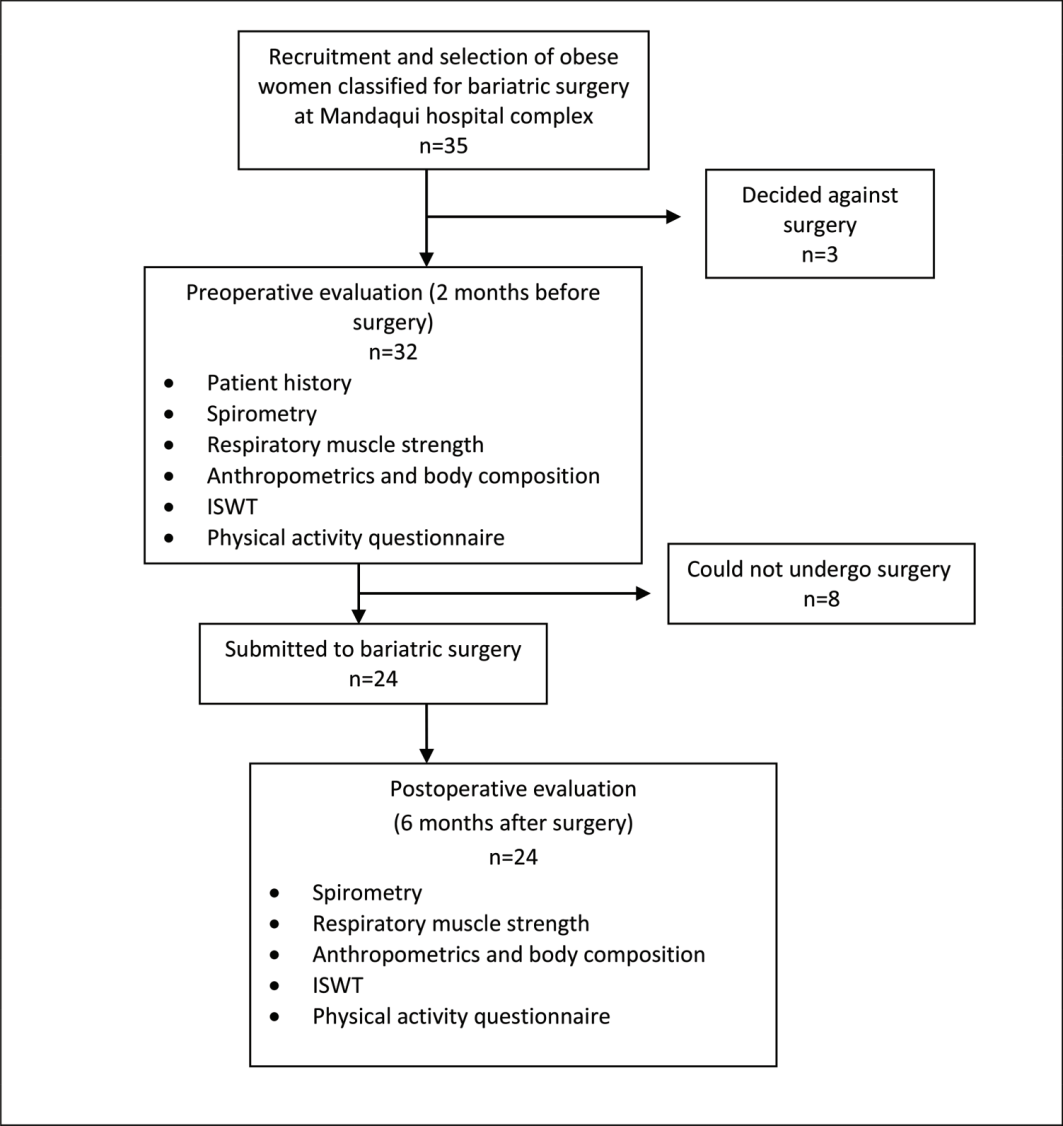
Flowchart of the study.

Among the 32 women with morbid obesity who met the inclusion criteria and were evaluated in the preoperative period, 24 participated until the end of the study; three decided against surgery, and two became pregnant and could not undergo surgery. Therefore, the results refer to the 24 women who participated in the evaluations before and after the operation (Figure 1).

After the organization and statistical treatment of the data, the results were arranged in tables and graphs for the different sets of variables analyzed: anthropometric variables, body composition, level of habitual physical activity, lung function, respiratory muscle strength and functional capacity. Table 1 displays the data related to anthropometric characteristics, body composition and level of physical activity before and after bariatric surgery.

Reductions in BMI, WC and NC were observed in the postoperative period. The percentage of excess weight also decreased significantly. Significant reductions in lean mass and fat mass (measured in kilograms) occurred, with a significant decrease in the proportion of fat mass and a significant increase in the proportion of lean mass in the postoperative evaluation. In contrast, there was no change in the level of physical activity determined using the Baecke questionnaire.

Table 2 displays lung function variables in liters (L) and the percentage of predicted (% pred) values.

When compared with the preoperative period, significant increases were found in both absolute values and percentages of predictive values for SVC, FVC and FEV_1_ in the postoperative period along with a significant increase in ERV in absolute values and a significant reduction in IC in absolute values. Conversely, no significant differences were found with regard to IRV in absolute values, FEV_1_/FVC in absolute and percentage of predicted values or MVV in percentage of predicted values.

Table 3 presents the results of analysis of respiratory muscle strength before and after bariatric surgery, as represented by MIP and MEP in cmH_2_O as well as in the percentage of predicted values based on the equation employed 25. The participants exhibited significant increases in MIP.

Table 4 shows the distance travelled on the ISWT in meters (absolute values and % pred values).

The mean distance on the ISWT was 329 meters; the predicted distance before surgery was 436 meters. Thus, the women walked less than expected (107 meters; 75% of the predicted distance). The mean distance following surgery was 401 meters, and the predicted distance was 503 meters, demonstrating that the women travelled less than expected (102 meters; 79% of the predicted distance). Nonetheless, the increase of 72 meters (22% of the initial distance) following weight loss was significant.

**Figure 2:**
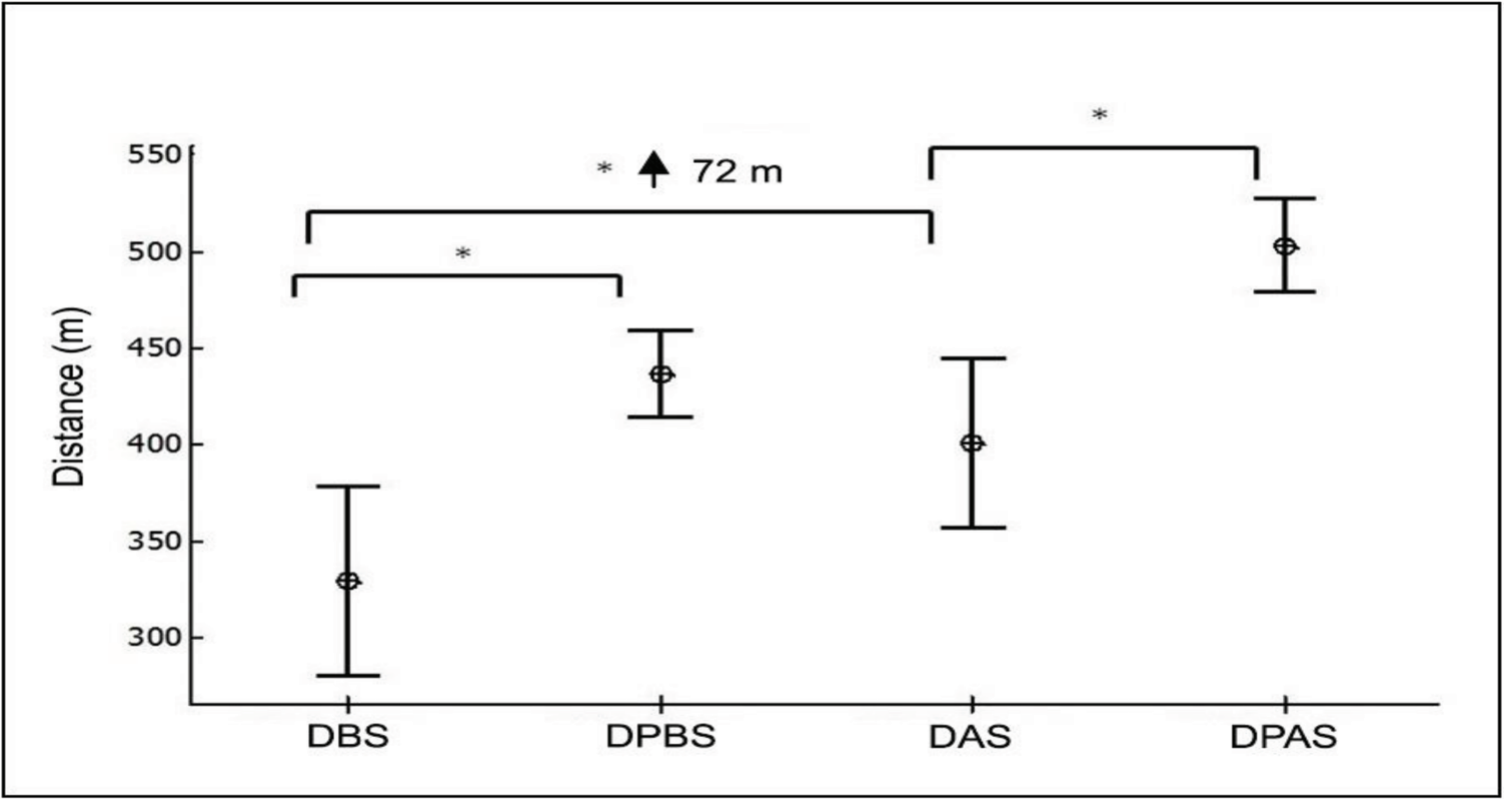
Distance on ISWT in preoperative and postoperative phases and predicted distance. DBS= distance before surgery; DPBS = distance predicted before surgery; DAS = distance after surgery; DPAS = distance predicted after surgery. *p≤0.05 compared DBS vs DPBS; DBS vs DAS and DAS vs DPAS.

Figure 2 illustrates the distance on the ISWT in the preoperative and postoperative periods as well as the respective distances predicted for individuals of the same age according to a previous study 19.

As shown in the figure, the distance (SD) travelled was greater after weight loss, and both distances before and after surgery were lower than the predicted distance for the sample under the conditions and with the anthropometric characteristics at the time of the tests.

Relationships among lung function, body composition and anthropometric variables were investigated based on the difference (d) between the preoperative and postoperative evaluations (Table 5).

Strong positive correlations were found between dIRV and dFM as well as between dIRV and dNC. Moderate positive correlations were found between dERV and dLM and also between dIC and dNC. Strong negative correlations were found between dIRv and dLM as well as between dERV and dNC. Moderate negative correlations were found betweendERV and dFM, between dFVC and dWC and between dFEV_1_ and dWC.

No correlations were found between the difference in respiratory muscle strength and any of the variables studied. No correlations were found between the difference in the distance on the ISWT and lung function variables, respiratory muscle strength, body composition or anthropometric variables.

## DISCUSSION

The results of the present study demonstrate that accentuated weight loss among women with morbid obesity in the first six months following bariatric surgery is associated with a significant improvement in lung function and functional capacity.

It should be stressed that the high values in the lung function variables SVC, FVC and FEV_1_ did not reflect any type of respiratory volumetric or flow abnormality in the preoperative period, as these variables and the FEV_1/_FVC ratio demonstrated no obstructive characteristics at baseline. The significant increase in lung volumes following weight loss reflects the principal effect of obesity on the reduction in lung volumes but not necessarily on airway obstruction. Excess adipose tissue causes mechanical compression on the diaphragm, lungs and thoracic cage, which can lead to decreased lung volume.

The 550-mL increase in ERV and the 500-mL reduction in IC in the postoperative period are in agreement with data described in the literature. ERV is notably lower in women with morbid obesity due to the upward displacement of the diaphragm caused by the compression resulting from an increased abdominal diameter due to excess adipose tissue. As a compensatory mechanism, IC increases to maintain vital capacity 31. However, these volumes tend to normalize following weight loss 32-34. Such findings are clinically relevant, as diminished ERV may determine a reduction in ventilation in the bases of the lungs, generating areas of pulmonary shunt due to the formation of atelectasis and sometimes resulting in hypoxemia 32. Thus, an individual may experience dyspnea and a low tolerance to exertion, progressing to limited functional capacity 32.

In addition to lung volumes and capacities, respiratory muscle strength is also of interest in the assessment of respiratory mechanics in obese individuals who are candidates for bariatric surgery. In a previous study involving women with morbid obesity, an increase in MIP was found following weight loss induced by bariatric surgery. The authors suggested that MIP is reduced in individuals with obesity due to the increase in the elastic load on the thoracic cage, thereby restricting expansion and making it difficult for the inspiratory muscles to overcome such a load. The authors also noted that muscle insertions may be in a state of mechanical disadvantage in individuals with obesity due to the deposition of fat mass in the thoracoabdominal compartment, which lowers the strength and efficiency of the respiratory musculature 35. Regardless, findings of the behavior of respiratory muscle strength in obese individuals are scarce and contradictory, as other authors have reported no abnormal values in the preoperative phase and a reduction in respiratory muscle strength following weight loss 36-38.

No abnormalities were found with regard to the MIP and MEP of the morbidly obese women compared with the values predicted in an earlier study 25. Nonetheless, MIP increased significantly by an average of 10 cmH_2_O following weight loss due to bariatric surgery. This finding is in disagreement with data from a previous study, in which the authors detected a reduction in MIP and no change in MEP among obese individuals who had undergone bariatric surgery 38.

Individuals with obesity have reduced functional capacity due to the increase in the BMI and consequent difficulty supporting their own body weight 19. Moreover, cardiopulmonary fitness is reduced in comparison to individuals in the ideal weight range due to heightened metabolic demand, low aerobic capacity and low tolerance to physical exercise 7-10.

Workload is strongly influenced by an increase in weight, and a higher BMI translates into a shorter distance travelled on the ISWT 19. In studies involving healthy individuals aged 50 to 85 years, a negative correlation was also found between BMI and distance travelled on the Six-Minute Walk Test 39. Although the women with morbid obesity in the present investigation demonstrated a significant increase in the distance travelled on the ISWT in the postoperative period, this finding was not correlated with a reduction in BMI.

The increase in functional capacity following weight loss induced by bariatric surgery was evidenced by the mean increase of 72 meters in the distance travelled on the ISWT. This result is in agreement with the findings of a previous study conducted by our research group that evaluated obese women before and after bariatric surgery; in that study, an increase of 72 meters in the walk distance was also found, though on the Six-Minute Walk Test 40.

Field tests are useful for the evaluation of functional capacity. As an incremental test, the ISWT resembles a stress test and therefore furnishes more information about aerobic fitness than does the Six-Minute Walk Test 41. In the present study, the obese women did not reach the predicted values 19, even after having lost weight. This finding is likely because despite weight loss in the postoperative period, 20 women (83% of the sample) continued to be obese during the postoperative evaluation (BMI >30 kg/m^2^). Thus, although initial weight loss was important for functional improvement, it was not sufficient to reach the predicted values. It is likely that these women will walk longer distances on the ISWT after further weight loss.

The participants did not undergo regular physical activity in the postoperative period. Individuals who receive a score of less than eight points on the Baecke questionnaire are considered sedentary 25. The scores in the present study were 3.87 and 4.01 points in the preoperative and postoperative periods, respectively.

The significant reduction in lean mass was possibly caused by a change in the absorption of nutrients due to the surgical procedure 42 as well as to the low-calorie diet generally prescribed in the postoperative period, which leads to proteolysis to meet the metabolic needs of the patient 43,44. These findings are in agreement with data described in a previous study 42 in which the authors analyzed body composition in obese individuals before and 30 days after bariatric surgery and found a mean reduction of 14 kg in weight, 5.2 kg/m^2^ in BMI, 9.7 kg in fat mass and 4.4 kg in lean mass after one month. The present findings are also in agreement with studies employing different methodologies 44-46.

The negative correlations between dWC and both dFVC and dFEV_1_ are similar to the data described by Wei et al. 47, who also evaluated obese individuals before and after bariatric surgery. These authors related improvement in lung function to weight loss and a reduction in intra-abdominal pressure caused by excess fat mass in this region, demonstrating that an increase in intra-abdominal pressure may account for pulmonary hypoventilation. Other researchers found that weight loss after bariatric surgery led to a significant reduction in patterns of ventilatory limitation associated with obesity 48.

Such clinical findings may guide new studies for this population and lend support to policies for the development of physical activity programs that can neutralize the loss of lean mass and cause these individuals to be physically active to prevent weight regain.

This study has limitations that should be considered. No male patients were included because none were being followed up at the Multidisciplinary Center for the Surgical Treatment of Morbid Obesity of Mandaqui hospital complex at the time the study was conducted. Moreover, some women at the center did not volunteer to participate in the study. A larger sample size would have increased the power of the findings.

The results of the present study demonstrate significant improvements in lung function and functional capacity in women with morbid obesity at six months after bariatric surgery, when a loss of excess weight, a reduction in anthropometric variables and altered body composition were observed. Despite such improvements, the level of physical activity still classified these women as sedentary, which may be related to a lack of habit in performing physical exercise. It is likely that the regular practice of physical activity in the first six months of the postoperative period would offer additional benefits to women with morbid obesity subjected to bariatric surgery.

## AUTHOR CONTRIBUTIONS

Campos EC was responsible for the recruitment of volunteers, data collection, data tabulation, bibliographic review, manuscript drafting and manuscript submission. Peixoto-Souza FS was responsible for the recruitment of volunteers, data collection, data tabulation, bibliographic review and manuscript drafting. Alves VC and Basso-Vanelli R were responsible for the bibliographic review and manuscript revision. Barbalho-Moulim M was responsible for the methodological orientation, bibliographic review and revision of the manuscript. Laurino-Neto RM was responsible for the recruitment of volunteers and manuscript revision. Costa D was responsible for the methodological orientation, bibliographic review, manuscript drafting and manuscript revision.

## Figures and Tables

**Table 1:** Anthropometric characteristics, body composition and level of physical activity of morbidly obese women before and after bariatric surgery.

	Before surgery	6 months after surgery	*p*
	n=24	n=24	
Age (years)	40±7	41±7	0.61
Anthropometrics			
Body mass (kg)	124.10±17.20	92.25±15.31	<0.0001*
BMI (kg/m^2^)	47.42±5.72	35.31±5.51	<0.0001*
WC (cm)	128.21±13.20	105.32±10.10	<0.0001*
NC (cm)	40.71±3.25	36.11±3.12	<0.0001*
W/HR	0.92±0.08	0.91±0.12	0.08
% Overweight	110.22±28.30	57.12±26.12	<0.0001*
Body Composition			
Lean mass (kg)	68.21±9.15	58.13±7.30	<0.0001*
Fat mass (kg)	56.23±10.11	34.12±10.11	<0.0001*
Lean mass (%)	55.12±3.15	63.14±6.15	<0.0001*
Fat mass (%)	44.12±3.15	36.12±6.13	<0.0001*
Level of physical activity			
PEL score	2.25 (1.75-2.5)	2.50 (2.25-2.81)	(<8)
LLA score	1.75 (0.75-2.25)	1.87 (1.00-2.25)	(<8)
QoL score	3.87 (3.12-4.5)	4.01 (3.25-5.00)	(<8)

BMI = body mass index; WC = waist circumference; NC = neck circumference; W/HR = waist-to-hip ratio; PEL score = Physical Exercise in Leisure; LLA score = Leisure + Locomotion Activities; QoL = quality of life questionnaire. *p≤0.05 compared to before surgery group.

**Table 2:** Spirometric variables before and after bariatric surgery.

	Before surgery	6 months after surgery	*p*
	n=24	n=24	
SVC (L)	3.37±0.67	3.53±0.69	0.01*
SVC (% pred)	92.27±12.40	99.86±13.25	<0.0001*
IRV (L)	1.93±0.72	1.67±0.51	0.22
IC (L)	2.98±0.84	2.49±0.54	0.04*
ERV (L)	0.41±0.20	0.96±0.55	0.01*
FVC (L)	3.16±0.52	3.45±0.69	0.02*
FVC (% pred)	91.54±12.03	97.41±13.51	0.003*
FEV_1_ (L)	2.64±0.46	2.89±0.61	0.02*
FEV_1_ (% pred)	92.81±13.03	98.71±15.41	0.007*
FEV_1_/FVC	83.81±4.03	83.51±3.34	0.74
FEV_1_/FVC (% pred)	100.36±5.55	101.21±4.55	0.4
MVV (% pred)	76.68±14.11	80.72±14.30	0.28

SVC = slow vital capacity; IRV = inspiratory reserve volume; IC = inspiratory capacity; ERV = expiratory reserve volume; FVC = forced vital capacity; FEV_1_ = forced expiratory volume in first second; MVV = maximum voluntary ventilation. *p≤0.05 compared to before surgery group.

**Table 3:** Static maximum respiratory pressure before and after bariatric surgery.

	Before surgery n=24	6 months after surgery n=24
MIP (cmH_2_O)		
MIP obtained	93.11±17.51	103.51±20.71*
MIP predicted (Neder^33^)	91.11±3.31	91.11±3.31
% MIP predicted	102%	113%
MEP (cmH_2_O)		
MEP obtained	91.11±14.31	98.11±20.11
MEP predicted (Neder^33^)	98.31±20.11	98.31±20.11
% MEP predicted	93%	100%

MIP = maximal inspiratory pressure; MEP = maximal expiratory pressure. *p≤0.05 compared to before surgery group.

**Table 4:** Distance traveled on ISWT before and after bariatric surgery.

	Before surgery n=24	6 months after surgery n=24
Distance (meters)	329±111	401±104*
% Predicted	75%	79%

* *p*≤0.05 compared to before surgery group.

**Table 5:** Correlations among lung function, body composition and anthropometric variables.

	dLM	dFM	dBMI	dWC	dNC
	r	r	r	r	r
dSVC (L)	0.38	-0.38	-0.29	-0.4	-0.5
dIC (L)	-0.5	0.2	0.14	0.16	0.60*
dIRV (L)	-0.73*	0.73*	0.33	0.28	0.76*
dERV (L)	0.63*	-0.63*	-0.29	-0.27	-0.74*
dFVC (L)	0.39	-0.39	-0.32	-0.68*	-0.45
dFEV_1_ (L)	0.43	-0.43	-0.28	-0.69*	-0.48

d = difference between preoperative and postoperative evaluation; r = correlation coefficient; dLM = difference in lean mass; dFM = difference in fat mass; dBMI = difference in body mass index; dWC = difference in waist circumference; dNC = difference in neck circumference; dSVC = difference in slow vital capacity; dIC = difference in inspiratory capacity; dIRV = difference in inspiratory reserve volume; dERV = difference in expiratory reserve volume; dFVC = difference in forced vital capacity; dFEV1 = difference in forced expiratory volume in first second; L = liters. *p≤0.05 in correlations.
